# Drone-based application of whale tags: A “tap-and-go” approach for scientific animal-borne investigations

**DOI:** 10.1371/journal.pone.0328037

**Published:** 2025-08-13

**Authors:** Daniel M. Vogt, Stefano Pagani, Zahrek Gonzalez-Peltier, Shane Gero, David F. Gruber, Robert J. Wood

**Affiliations:** 1 Harvard John A. Paulson School of Engineering and Applied Sciences, Harvard University, Cambridge, Massachusetts, United States of America; 2 Project CETI, New York, New York, United States of America and Dominica; 3 Department of Biology, Carleton University, Ottawa, Ontario, Canada; 4 Department of Natural Sciences, Baruch College and The Graduate Center PhD Program in Biology, City University of New York, New York, New York, United States of America; University of Saint Andrews, UNITED KINGDOM OF GREAT BRITAIN AND NORTHERN IRELAND

## Abstract

Deploying animal-borne suction-based tag devices on whales has been one of the primary tools used by researchers over the past several decades to gather high-resolution scientific information, such as bioacoustics, heart rate, dive depth, and body orientation. However, the process of successfully applying animal-borne tags is logistically challenging and requires substantial operator skill. Current methods apply tags by approaching the whale in a boat and adhering the tag via a long extension pole. In this study, we explore an alternative approach to apply animal-borne suction-based tag devices using First Person View (FPV) racing drones. These drones have been specifically adapted to withstand exposure to seawater, allowing them to operate effectively in marine environments. The drones are equipped with a custom interface, allowing to release the tag when it is applied on the whale’s back. In this study, we present the development of the delivery drone as well as tag deployment techniques. The proposed method was demonstrated on sperm whales (*Physeter macrocephalus*) off Dominica, resulting in fast deployment time (one minute and fifteen seconds on average) and a relatively high deployment success rate (over 55 %). In addition, the presented deployment process offers a less invasive technique for tagging, as boats are not needed for close approaches. These methods also serve as a framework to enable future development of more automated solutions to apply the tag on exact anatomical targets with controlled initial adhesion pressure and without manual operation.

## Introduction

Over the past several decades, human understanding of whale behavior has greatly increased, largely due to the use of on-whale tags as a primary research tool. The deep-diving behavior of sperm whales makes them difficult to study by visual observation and hence on-whale tags have been one of the key tools that have enabled insights into their physiology and behavior [1, 2]. Technology advances in adjacent fields have helped to improve the fidelity and diversity of data collected by such tags. For example, while prior devices only collected sparse GPS positions and dive profiles based on pressure sensors, more modern behavioral tags are able to collect high resolution audio and movement data in addition to position and depth [3–5].

**Fig 1:**
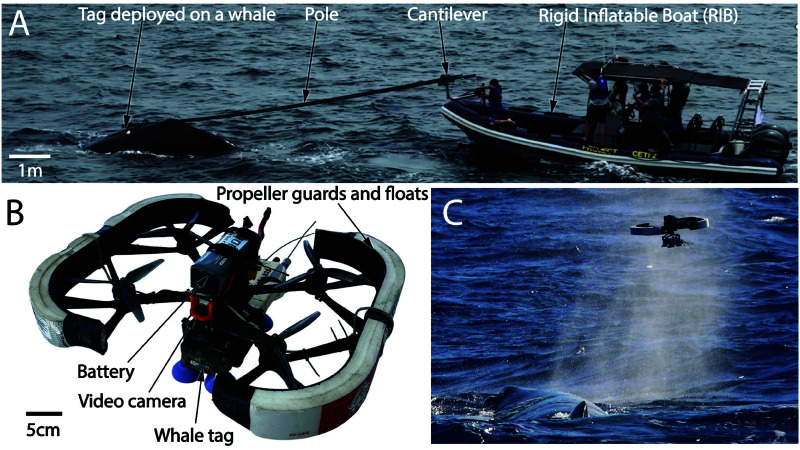
Approaches for tagging. A: An example of pole tagging. B: The racing drone used for tagging. C: A racing drone just prior to tagging.

Deploying these tags on the backs of whales in their natural environment is a challenging task. First, the whales need to be located. Second, the whales need to be carefully approached with the tag. For deep diving species, such as sperm whales, their surface time is only a fraction of their dive time (approximately 45 minutes dive time for 8-10 minutes at the surface [6]). Thus locating a whale and getting a boat into position to tag during a surface interval requires extensive preparation and coordination. Third, the final approach of adhering the tag to the whale requires considerable skill and care. With pole tagging (Fig 1A), the most widely used technique over the past decade [7], the tag is attached at the distal end of a long pole. Once within a few meters of the whale, the tag is applied manually by manipulating the pole to bring the tag in contact with the whale. The whole pole tagging operation takes approximately three to five minutes (from the initial sighting of the whale to deploying the tag on it).

Although pole tagging is effective and stands as the state of the art for tag deployment, it has several limitations. First, it requires a boat to come in close proximity with the whale. This is not only challenging to do because of the short time window offered by the whales when they surface, but can also potentially impact their behavior because of an unusual presence (i.e., the approaching boat) and engine noise. Second, specific equipment is required on the boat to mount the pole. This is not adaptable to any boat: this method requires both specialized infrastructure and personnel (i.e., a captain and pole tagger) to be successful.

Due to these limitations, researchers have investigated alternative methods for deploying suction cup based behavioral tag on a whale using drones [8]. One such method, demonstrated in [9], involves flying a drone capable of carrying a substantial payload above a whale and passively dropping the tag onto it. During the drop, a temporary fin remains attached to the tag to ensure that the suction cups remain oriented downwards upon contact. This fin releases upon impact with the whale. Other methods have been explored including shooting tags (and other packages) with a crossbow or air gun [7, 10] to attach by barbs. However, we restrict the discussion in this paper to suction-based adhesion systems since we aim to impart minimal damage to the animals we study.

In this paper, we introduce an inexpensive and rapid method for deploying tags using small drones. Our approach involves modifying a FPV racing drone (Fig 1B and 1C) specifically for whale tagging purposes. By harnessing the speed and agility of these drones, we enhance the tagging process by reducing the need for a tagging vessel in close proximity to the whale and by reducing the tagging process time by at least a factor of three.

## Materials and methods

### Drone platform

The drone utilized in this study is built upon a 7” frame (Source one, TBS Avionics Co Ltd, Hong Kong SAR, China). This configuration is commonly used within the FPV racing and freestyle community due to its resilience to impacts and flight maneuverability. A comprehensive breakdown of readily available components is provided in S1 Table. It is important to note that this list represents the essential components necessary for the drone’s operation and excludes accessories such as a LiPo battery charger or other specialized tools required for drone maintenance and operation.

This drone is typically operated in *Acro Mode* where the pilot directly controls the drone’s angular rates. It falls upon the remote pilot to continuously adjust the flight controls, as the drone does not auto-correct itself and enter a stable hover when the pilot lets go of the controls, unlike conventional off-the-shelf drones (e.g., drones from *DJI*). While there is initially a steeper learning curve to pilot this type of drone using this mode, it opens the door to more advanced flying capabilities, which are not possible with position-holding flight modes common in commercial drones.

A second mode, called *Angle Mode*, can be used during training and as a back up in emergency situations (e.g., a loss of the video link) to keep the drone flying horizontally while recovering from the fault.

### Drone waterproofing and floatability

To effectively tag whales, the drone platform was modified in several ways. First, a key limitation of commercial drones is that they are susceptible to water ingress. To protect against damage from marine conditions, we developed a method to waterproof nearly all the electronic components to withstand splashes, and for some components, complete immersion in seawater.

**Fig 2:**
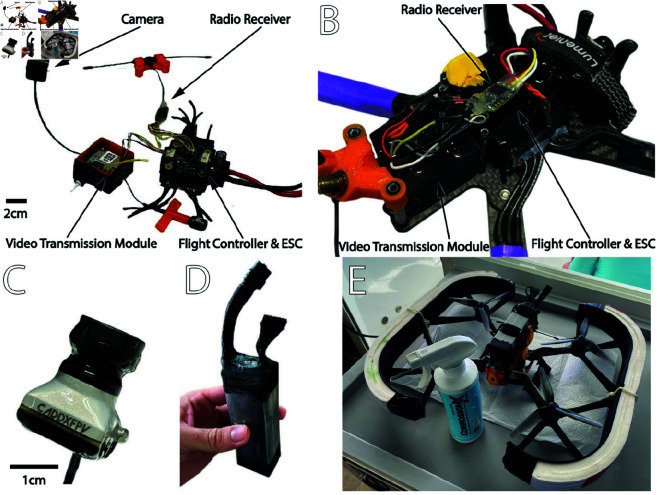
Main waterproofing steps. A: The Flight Controller and the Video Transmitter Unit prior to potting with thermally conductive epoxy. B: Flight Controller and the Video Transmitter Unit after potting. C: Adding liquid rubber around the camera (except the lens; the lens cap was left on). D: Coating the battery with liquid tape. E: Treating the motors with corrosion prevention compound.

1. The **Flight Controller** and the **Video Transmitter Module** (Fig 2A and 2B) were cast in thermally conductive epoxy (832TC-2L, MG Chemicals, Burlington, Ontario, Canada) to sink heat generated during operation. If waste heat is not properly removed, there is a risk of permanent damage to these components, including during a typical flight. Note that there are buttons on these components to either bind the video link to goggles or start the flight controller in *boot mode*. In order to remain functional, these buttons were removed prior to potting and replaced by wires that can be shorted externally to emulate a button push. In addition to the buttons, the USB connection remained accessible for firmware updates and to update configuration settings. To do so, a USB-mini connector treated with grease (111 O-Ring Silicone Lubricant, Dow Corning, Midland, MI, USA) was left connected in the device while potting. Once cured, the temporary connector was removed, and the cavity created was then be filled with a sculptable dental silicone compound (Elite HD+, Zhermack, Badia Polesine, Italy) to maintain waterproofing. Finally, the camera (Fig 2C) was coated everywhere except on the lens with liquid rubber (Flex Seal Liquid, Greensboro, GA, USA).
2. The **Radio Receiver** (Fig 2A and 2B) was waterproofed using transparent heat shrink tubing wrapped around the receiver unit and subsequently injecting a sealant (Clear All Purpose Silicone Caulk, General Electric, Boston, MA, USA). Similar to the Flight Controller and the Video Transmitter Module, the binding button was removed and replaced by external wires.
3. The **Battery** (Fig 2D) was waterproofed by brushing liquid tape (Plasti Dip International, Blaine, MN, USA) on the front and back extremities to avoid water infiltration inside the battery. Liquid tape was also be applied to the battery leads until reaching the battery connector (for both the main battery lead and the balancing port). A 3D-printed cap made out of flexible plastic (TPU Ninjaflex, Ninjatek, St. Manheim, PA, USA) was used to cover the balancing port. Each connector was stuffed with dielectric grease before being used on the drone.
4. The **Motors** (Fig 2E) are already waterproof due to the nature of the coated windings. However, they must be regularly treated with a corrosion prevention and control compound (CorrosionX Aviation 80103, Corrosion Technologies, Garland, TX, USA). If exposed to sea water, the motors must first be rinsed with fresh water and dried with compressed air.
5. Finally, all the original **Frame Screws** are replaced with stainless steel equivalents.

After waterproofing, another safety measure is implemented on the drone to enhance the safety of the tagging operation. This is done by installing custom 3D printed (Nylon 12, Fuse, Formlabs, Somerville, MA, USA) guards around each arm extremity to minimize the likelihood of the whale coming into contact with the rotating propellers.

Lastly, precautions must be implemented to address the eventuality of the drone falling into the water and sinking. Given that the whale tags are already positively buoyant by nature (they need to float to be recovered at the end of the deployment), efforts can focus on making the drone positively buoyant. For this purpose, several layers (Volume: $1.69\times 10^{-3}\;{\text{m}}^{3}$) of polyethylene closed cell foam (93565K99, McMaster-Carr, Elmhurst, IL, USA) with adhesive backing are applied around the prop-guards. This foam has a density of $32\;{\text{kg/m}}^{3}$ and is depicted in yellow in Fig 4. It provides up to 1.64 kg of flotation, exceeding the weight of the waterproofed drone alone (1.6 kg). In addition to buoyancy, this layer of foam also serves as soft bumper in case the drone is not perfectly aligned with the whale’s skin during deployment.

With these modifications, the drone is sufficiently waterproof and safe for operation in close proximity to whales. Our study has monitored these whales behaviorally and photographically for many years and all of the tagged animals have been encountered after these tagging events with no evidence of injury or changes to their behavior.

### Tag holder and on-whale adhesion

**Fig 3:**
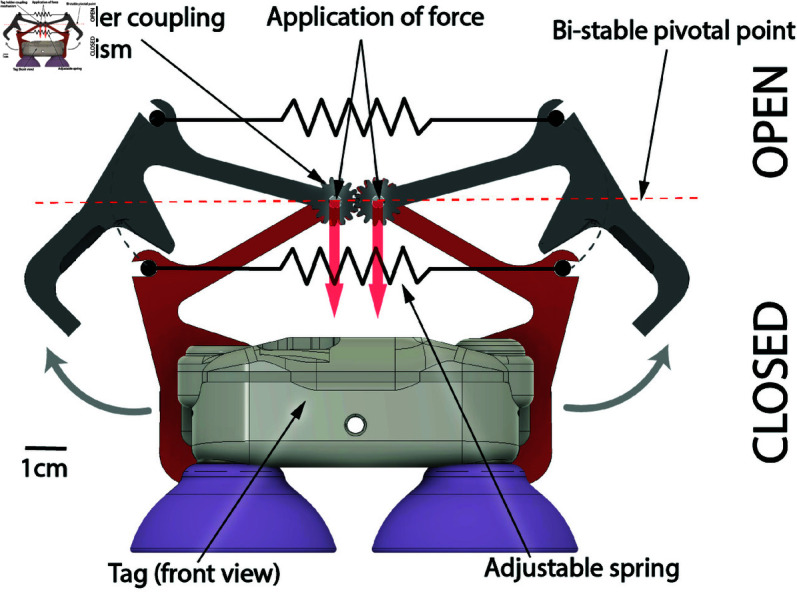
Tag holder concept. A bi-stable mechanical design (in red) is intended to release the tag when a downward force is applied (i.e., from impact when landing on the whale).

To deploy a tag on a whale, a specialized mechanism is required to securely hold the tag while the drone is in flight and release during deployment on the whale. For this purpose, a bi-stable mechanism was designed as shown in Fig 3. This mechanism comprises a tag holder [11] that firmly grips the tag when closed. Upon vertical downwards pressure, the tag holder will open until the spring exceeds the bi-stable pivot point which switches the tag holder to an open configuration. Additionally, both sides of the tag holder are coupled with a gear to ensure stability around the roll axis for any tag holder opening angle.

By adjusting the tension of the spring using cable ties, it becomes possible to fine-tune the force required for the tag holder to open and release the tag. Typically, this force is set to 30*N* which corresponds to the tag holder release force needed when the suction cups have been firmly pressed on the whale.

For the custom-made suction cups and the tag used, the required work to ensure adhesion is *W*_*TagAdhesion*_ = 1.7*J* (S1 Appendix). From an energy conservation standpoint, the potential energy of the drone and tag of mass $m_{Drone\&tag}$ hovering at a height *h* and subject to gravity *g* can be expressed at $E_{Potencial}=m_{Drone\&tag}\times g\times h$. The height required can then be expressed as $h=\frac{W_{TagAdhesion}}{m_{Drone\&tag\times g}}=75.3\times 10^{-3}m$.

In practice, the drone and tag are triggered to fall from a height of approximately *h*_*Practice*_ = 0.5*m* to account for other factors such as drag, the fact that the propellers continue rotating and stabilizing the drone during the fall, and angular misalignment between the suction cups and the whale’s skin.

### Drone specifications and performance

**Fig 4:**
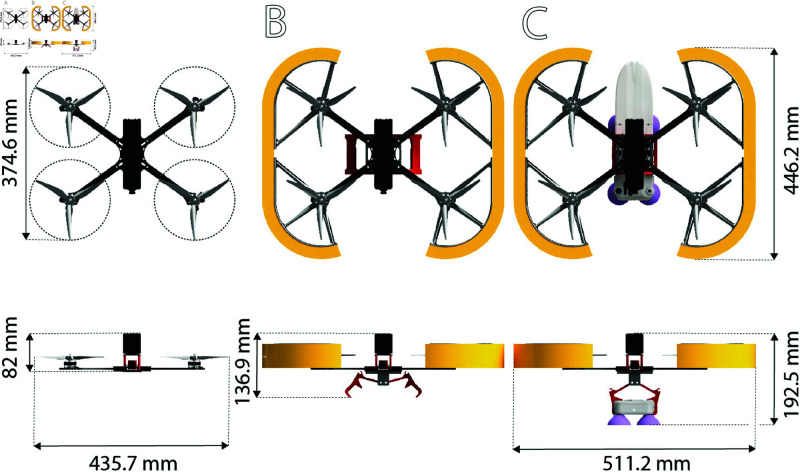
Drone configurations. A: Core drone. B: Waterproofed Drone. C: Waterproofed drone carrying a tag.

The main drone specifications are summarized in Table 1 for various configurations (shown in Fig 4). These parameters are important to keep in mind when defining the flight range (with or without a tag). Regardless of the configuration, the drone’s maximum thrust is 10.26 kg [12], which is 4.5× more than its heaviest configuration and results in reasonable headroom for additional payload and maneuverability.

**Table 1:** Comparison of drone specifications. The weight of the tag used in these estimates is 0.7 kg. The estimated flight time is based on the use of a 6S (22V averaged) 2900mAh battery and [12]. Note that the real world flight times are approximately 30% less than the theoretical values due to parameters such as video transmission (up to 9 W), drone design (motor arm and propeller guard impeding on the air flow), the flight environment (wind, air temperature, and density), and the drone operation (taking off or cruising towards the whale).

Parameter	Core Drone	Water-proofed Drone	Water-proofed Drone carrying a tag
Weight	1.2 kg	1.6 kg	2.3 kg
Theoretical power to hover [12]	243 W	318 W	483 W
Theoretical maximum flight time (hover)	15.8 min	12 min	7.9 min

### Description of the tagging method

**Fig 5:**
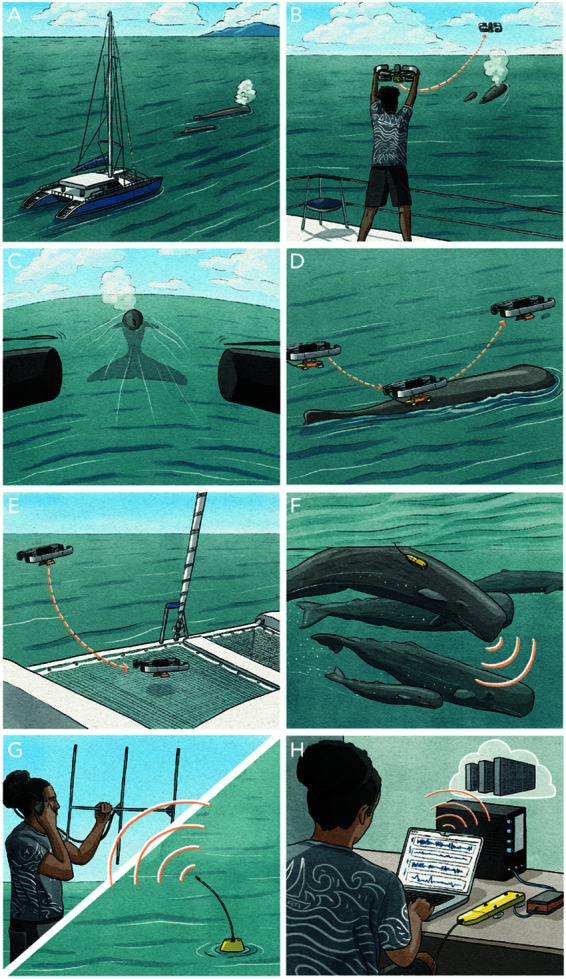
Tagging method overview. A: Looking for whales. B: Launching the drone from the boat. C: Aligning the drone behind the whale prior to tag deployment. D: Tag deployment on the whale via “tap-and-go”. E: Drone return on the boat. F: Data recording on the whale. G: Tag retrieval. H: Data offload and tag reconditioning for the next deployment.

The complete process of whale tagging is described in Fig 5. The drone can be hand-launched from a variety of platforms, including larger vessels such as a 40ft sailboat (Fig 5A and 5B) or smaller rigid hull inflatables. After take off (Fig 5B), the pilot must be able to locate the target whale in their FPV headset and/or with the support of other observers onboard. The pilot can then align the drone with the whale. This is aided by the propeller guards creating a directed field of vision (Fig 5C). Deployment is achieved by the drone descending to the back of the whale and making contact with sufficient force to release the tag from the bi-stable tag holder (Fig 5D).

**Fig 6:**
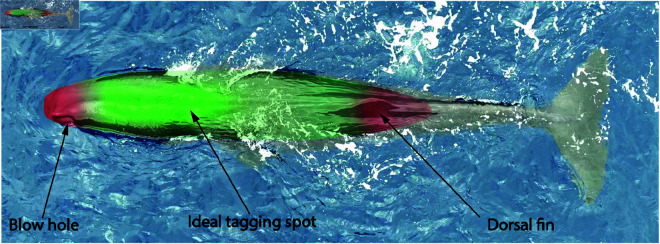
Ideal tag deployment location. The ideal deployment spot is between the dorsal fin and the blow hole, with a preference towards the front to be closer to the spermaceti organ.

The ideal tag placement on the back of the whale is between the dorsal fin and the blow hole (Fig 6). A placement towards the front is preferable as it will result in better audio quality as the tag will be in closer proximity to the spermaceti organ. However, one must take care not to place the tag too far forward in order to avoid the blow hole – a sensitive respiratory orifice and must not be obstructed. Deploying too close to the dorsal fin can also be more challenging as the whale’s dorsal curvature is generally greater there.

Once the tag is successfully placed, the drone can then come back to the boat for landing or collect video footage post deployment (i.e., for subsequent behavior annotation, to confirm deployment is secure, and document tag orientation and location on the whale). Fig 5E depicts the drone returning to land on the host vessel.

Data is then recorded on the whale (Fig 5F) for the time of the deployment, which can vary between a few hours to a few days. When the tag detaches from the whale, several technologies can be used to locate it, such as VHF telemetry (Fig 5G) or satellite messaging [13]. Once recovered (Fig 5H), data can then be offloaded from the tag and the tag can be reconditioned for the next deployment.

The tag used for this research is a *CETI tag*. A typical tag measures 110×50.6×309mm (without antennas) and weighs 0.7kg. This research has been conducted under the Dominica Fisheries Research Permit # LS 27-200-21 and Harvard IACUC Protocol ID # 21-02-379-1.

## Results and discussion

### In-field whale tagging

Drone-based tagging constitutes one of the most effective methods to deploy tags on sperm whales (*Physeter macrocephalus*) for large scale data gathering, ultimately providing an invaluable data stream for Project CETI [14] (CEtacean Translation Initiative) and related marine mammal research.

**Fig 7:**
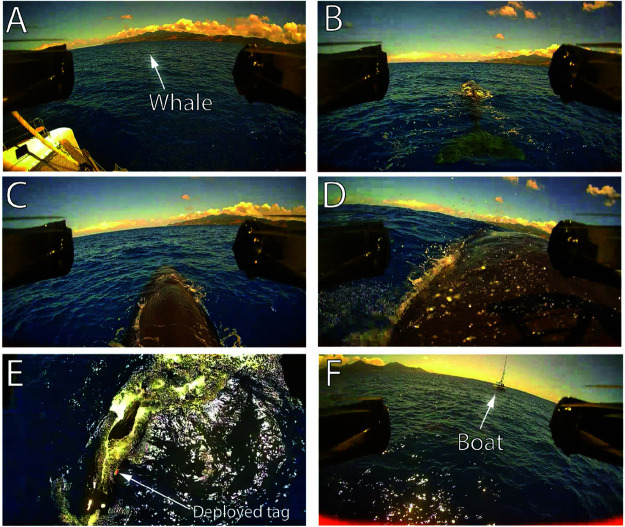
In-field deployment. A: Drone launch from the boat. B: Drone alignment behind the whale. C: Passing the dorsal fin and synchronizing with waves breaking over the whale. D: Descent and tag adhesion to the whale. E: Post-deployment verification that the tag is stuck on the whale. F: Return to the boat.

Fig 7 and S1 Video show an example of a successful tag deployment that occurred during a field expedition on March 2024. The tag adhered on the whale (named *Fingers*) for three hours before the timed released was triggered and the tag was recovered.

The whole tagging operation (from sighting of the whale at the surface to landing on the boat after deployment) took less than seven minutes, which can be broken down as follows:

- Boat to whale travel, approximately 100m (40 seconds);
- Alignment with the whale and tag deployment (20 seconds);
- Post-deployment behavioral observation and documentation of tag orientation and location; as well as confirmation that the tag is successfully adhered on the whale (*Optional*, 5 minutes); and
- Return to the boat (40 seconds).

During a recent tagging expedition using this deployment method, a total of twenty tagging attempts were made (S1 Table). Our criteria for successful deployment requires that the tag remains adhered to the whale for at least one shallow dive following the deployment. It is possible that the tag will detach from the whale after one dive, but that is not necessarily be correlated to the drone deployment method. Other factors can affect how long a tag will stay on a whale such as: the tag design, suction cup design, whale skin conditions under the suction cups, interactions with other whales, breaching, etc. Overall we observed a success rate of 55% and an average deployment time (take-off to whale tagging attempt) of one minute and fifteen seconds.

Importantly, our research vessel was often hundreds of meters away from the focal whale we intended to tag; it is unlikely that a traditional boat used for pole-based deployment would be able to arrive in time (i.e., before the whale dives) for traditional pole tagging to be successful.

### Limitations

**Fig 8:**
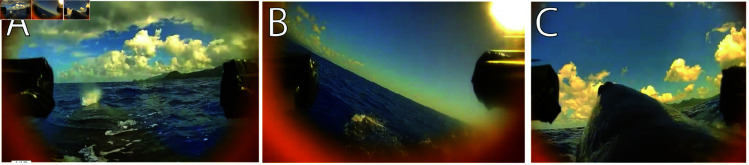
Limitations of the proposed tagging method. A: The presence of water on the targeted adhesion spot of the whale can impede on suction cup adhesion. B: Windy conditions can make the flight challenging and requires wind compensation, affecting deployment accuracy. C: Hovering too long just above the whale will likely cause the whale to notice the drone and camber its back, resulting in the target deployment spot being underwater.

Despite the success of the proposed drone tagging method compared with pole tagging, it can be subject to some limitations which are summarized in Fig 8.

First, similar to pole tagging, the timing of drone descent is key to make sure that the targeted surface on the whale is not under water due to wave action (Fig 8A). Otherwise, the suction cup will not engage fully because of the presence of an incompressible fluid. Additionally, sea conditions above sea state three (with a wave height of 0.5 to 1.25 meters) will increase the wave coverage over the whale and limit the deployment window. Nonetheless, it was our experience that we were able to deploy tags by using this method which we would not otherwise have been possible using the more traditional hand-operated pole technique. We speculate that this is due, in part, to windy conditions that cause the focal whale dive in response to the noise created by waves hitting the boat’s hull. Thus, by removing the vessel from the deployment process we are able to deploy any time it is possible to fly.

Another limitation similarly stems from windy conditions (above 15-20 knots), which can lead to challenging flight conditions, where the pilot must tilt the drone to compensate for the wind (Fig 8B) and will induce sideways drift when descending to deploy a tag on the whale. This is a scenario in which the agility of racing drones provides a clear benefit over commercial drones that are restricted to station-keeping flight modes when working in challenging near-water conditions.

Finally, with current Lithium Polymer (LiPo) battery technology, the flight time is limited to approximately ten minutes (further reduced to less than six minutes when carrying the tag). Hovering directly over the whale prior deployment should be limited to a few seconds, as the whale might start noticing the drone [15] and abruptly camber its back under the water level (Fig 8C) which will make the deployment challenging.

## Conclusion

In this paper, an innovative “tap-and-go” whale tagging method using racing drones is demonstrated. This method offers a less intrusive tag application process compared to pole tagging, where a close approach to the whale by boat is needed. The “tap-and-go” method is also very quick (less than two minutes in the above example) and has a success rate of 55%, leading to less invasive tagging by removing the need of a nearby boat during the tagging operation, and ultimately leading to greater data throughput.

In contrast to tag drops from drones flying at higher altitudes [9], this low-altitude flying method offers the advantage of precise timing of the tag’s impact with the whale, thereby minimizing interference from water coverage caused by waves. Additionally, passive drops from higher altitudes are more susceptible to wind drift during the period between the drop and impact.

Finally, drone-based tagging is also a cost effective method as the primary drone components cost approximately $1300. The drone can be launched from virtually any type of boat and the high payload capacity can accommodate many kinds of tags (only requiring modification of the tag holder).

Although these drones require advanced piloting expertise, this can be alleviated via flight simulators to develop the necessary skill. Furthermore, future efforts will focus on expanding the proposed method to greater autonomy, in particular for the more skill-intensive portions including target location identification and to control flight during the critical attachment phase of the deployment.

Like other tagging methods, drone-based tagging has limitations such as sensitivity to sea conditions (wind and swell) and whale behavior. Overall, we have demonstrated that this method is faster and more efficient in terms of logistics compared to more traditional pole tagging. Future directions can focus on the use of machine learning tools to develop controllers to automate the tagging process, making sure the tags are applied to the appropriate location with sufficient pressure.

## Supporting information

S1 Video In-field deployment. (MP4)

S1 Appendix Energy required for suction cup adhesion. (PDF)

S1 Table Bill of material for the drone. (PDF)

S2 Table In-field deployment attempts. (PDF)
