# Supplementary material for: Drone-based application of whale tags: A “tap-and-go” approach for scientific animal-borne investigations
Source: PLoS One. 2025 Aug 13;20(8):e0328037. doi: 10.1371/journal.pone.0328037 (PMC12348971; doi:10.1371/journal.pone.0328037)
Supplement: S1 Appendix — (PDF) [file pone.0328037.s002.pdf]

S1 Appendix: Energy required for suction cup adhesion

The work needed to adhere a suction cup is determined as follows:

$$W_{SuctionCup} = \int_{x_1}^{x_2} F_{Adhesion}(x)dx$$

With $F_{Adhesion}(x)$ the force applied to the suction cup over a distance x.

To estimate that value, a material tester (Instron 5544A, Instron, Norwood, MA 02062, USA) was used to measure the force and displacement data.

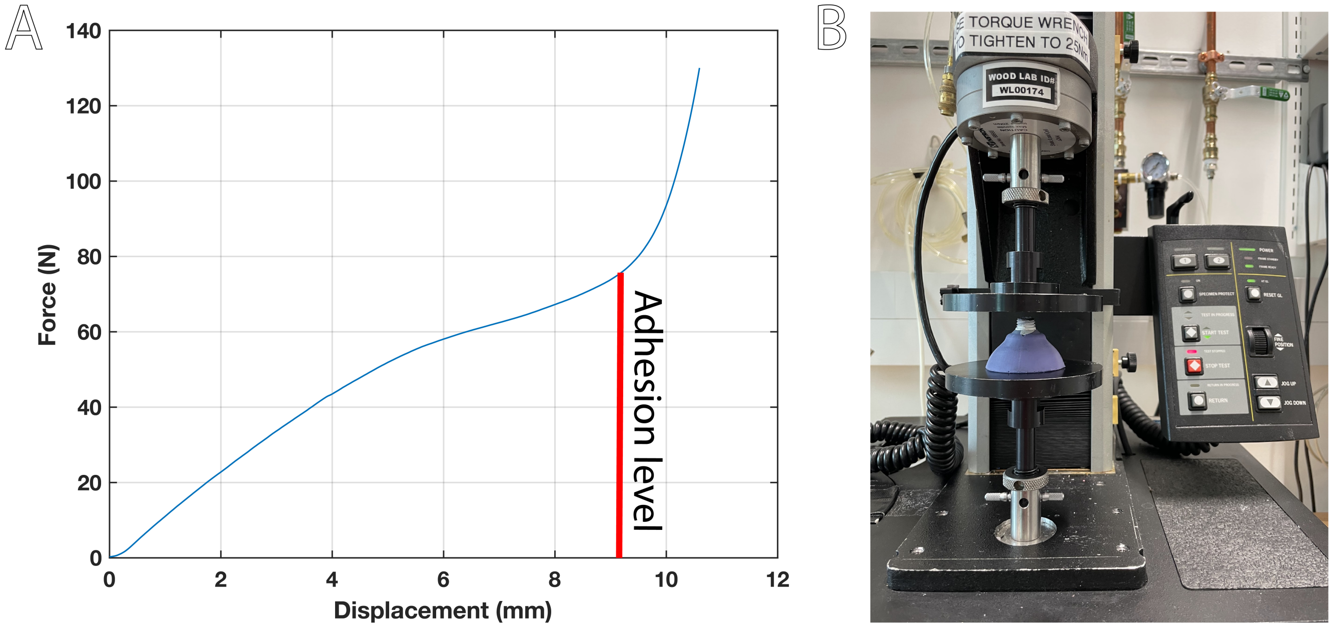

Figure 1: **Experimental calculation of the work to adhere a suction cup.** A: Force vs displacement. B: Experimental setup.

The work is given by calculating the area under the curve, and equals to $W_{SuctionCup} = 2J$ for one suction cup, or $W_{TagAdhesion} = 8J$ for the whole tag.
