# Supplementary material for: Drone-based application of whale tags: A “tap-and-go” approach for scientific animal-borne investigations
Source: PLoS One. 2025 Aug 13;20(8):e0328037. doi: 10.1371/journal.pone.0328037 (PMC12348971; doi:10.1371/journal.pone.0328037)
Supplement: S1 Table — (PDF) [file pone.0328037.s003.pdf]

S1 Table: Bill of Material for the drone

**Component**	**Description**	**Quantity**	**Cost ($)**
Frame	Pyrodrone Source One 7" Long Range	1	29.97
Motors	T-Motor F100 2810 LR Cinematic Motor 1350kV	4	35.99
Propellers	HQProp DP 7X3.5X3 PC Propeller	4	1.00
Flight Controller & ESC	MOTOR F7 HD (Betaflight firmware Version 4.4.2) & F55A PRO II	1	137.90
Battery	Lumenier 2900mAh 6S 120c CineLifter LiPo Battery	1	103.99
Radio Transmitter	RadioMaster TX16S MKII MAX 2.4GHz 16CH	1	269.99
Radio Receiver	TBS Crossfire Nano Rx (SE) w/ Immortal T V2 Antenna	1	34.99
Video Transmission Module	Caddx Polar Micro Digital FPV Vista Camera Kit (Firmware V1.00.0608)	1	139.99
FPV Goggles	DJI FPV Goggles V2 (Firmware V1.00.0606)	1	349.00
**Total Cost**			**$1212.89**

Table 1: Bill of materials for key drone parts. The prices were updated on March 19th, 2024.
