# Supplementary material for: Drone-based application of whale tags: A “tap-and-go” approach for scientific animal-borne investigations
Source: PLoS One. 2025 Aug 13;20(8):e0328037. doi: 10.1371/journal.pone.0328037 (PMC12348971; doi:10.1371/journal.pone.0328037)
Supplement: S2 Table — (PDF) [file pone.0328037.s004.pdf]

S2 Table: In-field deployment listing

Date	Adhesion Status	Take-off to deployment time
16:40 UTC, July 5th, 2023	Attached	3m24s
16:50 UTC, July 6th, 2023	Attached	1m43s
14:34 UTC, July 9th, 2023	Detached	1m15s
15:07 UTC, July 9th, 2023	Attached	59s
14:57 UTC February 28th, 2024	Attached	43s
16:29 UTC February 28th, 2024	Attached*	60s
17:01 UTC February 28th, 2024	Detached	57s
18:02 UTC February 28th, 2024	Detached	51s
19:34 UTC February 28th, 2024	Detached	1m5s
18:45 UTC February 29th, 2024	Attached	1m18s
17:58 UTC March 1st, 2024	Detached	3m12s
19:00 UTC March 1st, 2024	Detached	1m43s
21:32 UTC March 1st, 2024	Detached	30s
22:33 UTC March 1st, 2024	Detached	1m20s
14:32 UTC March 2nd, 2024	Attached	54s
17:33 UTC, March 2nd, 2024	Attached	60s
19:17 UTC, March 2nd, 2024	Attached	36s
20:10 UTC, March 2nd, 2024	Attached	33s
15:32 UTC, March 4th, 2024	Detached	33s
16:01 UTC, March 4th, 2024	Attached	1m15s

A successful deployment is defined as a tag adhering on a whale following the impact. It can happen that the tag will then detach from the whale after a shallow dive(denoted by a '*'), but that will not be relevant to the drone deployment method. Other factors can affect how long a tag will stay on a whale such as: tag design, suction cup design, skin condition under the suction cups, interactions with other whale, breaching, etc...

From the data in the table, 11 deployments out of a total of 20 attempts (55%) successfully stuck on the whale. On average, it takes *1m15s* from take-off from the boat to the deployment attempt on a whale.
